# Evaluation of camelina seed pods as a novel feed ingredient for ruminants: nutritional value, fermentation characteristics and nutrient digestibility

**DOI:** 10.1016/j.vas.2025.100477

**Published:** 2025-07-11

**Authors:** Alireza Jolazadeh, Ayoub Azizi, Afrooz Sharifi, Fatemeh Memarzadeh, Nazanin Fallali, Elham Imani-Rad

**Affiliations:** a Department of Animal Science, Safiabad-Dezful Agricultural and Natural Resources Research and Education Center, Education & Extension Organization (AREEO), Dezful, Iran; b Department of Animal Science, Faculty of Agriculture, Lorestan University, Khorramabad, Iran; c Animal Science Research Department, Khuzestan Agricultural and Natural Resources Research and Education Center AREEO, Ahvaz, Iran

**Keywords:** Agricultural waste, Alternative ingredient, Fermentation characteristics, Sheep

## Abstract

This study aimed to assess the chemical composition and nutritional value of camelina seed pods (CSP), as well as to investigate how different inclusion levels influence diet digestibility and *in vitro* rumen fermentation characteristics. Camelina seed pods were analyzed for their chemical composition and nutritional value, and the results were compared to those of wheat straw (WS) and alfalfa hay (AH). The study further investigated the impact of increasing CSP inclusion levels (0, 80, 160, and 240 g/kg DM) as a forage replacement in the diet on *in vitro* rumen fermentation parameters. Results revealed that CSP had the highest organic matter (OM) content and the lowest acid detergent fiber (ADF) levels (*P* < 0.05). Its crude protein and neutral detergent fiber (NDF) content were intermediate between WS and AH. Furthermore, CSP outperformed both WS and AH in terms of ether extract and non-fiber carbohydrate content (*P* < 0.05). Compared to WS, significantly greater gas production (GP) was observed for CSP at 16, 24, 36, 48, and 72 h of incubation, along with increased total GP and greater GP potential (b). After 24 h of incubation, higher fermentation parameters including *in vitro* DM (IVDMD) and OM (IVOMD) disappearance, estimated metabolizable energy (ME), microbial protein production (MP), ammonia-N (NH₃-N) concentration, pH, and short-chain fatty acid (SCFA) concentration were observed for CSP compared to WS, but lower than those for AH (*P* < 0.05). Nevertheless, all GP parameters for CSP remained lower than those observed in AH. Increasing CSP supplementation up to 16 % resulted in a linear improvement in IVOMD, estimated ME, and SCFA production compared to other supplementation levels (L, *P* < 0.05). The activity of fibrolytic enzymes, including CMCase, increased linearly (L, *P* < 0.05) with higher levels of CSP in the diet. In summary, the results of this study demonstrate that CSP enhanced *in vitro* gas production, fermentation characteristics, and nutrient digestibility in comparison to WS. Moreover, several of these parameters were found to be comparable to those of AH.

## Highlights

• We evaluated camelina seed pods as a novel feed ingredient for ruminants’ nutrition.
• CSP outperformed WS in terms of GP, nutrient digestibility and fermentation efficiency, and exhibited several fermentation parameters comparable to those of AH.
• Camelina seed pods possess favorable chemical and nutritional characteristics, positioning it as a viable alternative forage source in ruminant diets.

## 1 Introduction

The rising global demand for livestock products has driven the search for sustainable and cost-effective feed alternatives that do not compete with human food supplies. With over 60–70 % of livestock production costs attributed to feed (Salami et al., 2019), utilizing agricultural by-products for animal feed has become increasingly important, especially in light of limited resources such as arable land and water. Incorporating agricultural residues into livestock and poultry diets offers a viable strategy for reducing costs (Kazemi, 2025) and mitigating environmental pollution (Shah et al., 2025). These residues, sourced primarily from agricultural production and processing, provide opportunities to enhance productivity and conserve resources. Additionally, the strategic use of nutritionally rich agricultural by-products helps optimize resource utilization, alleviating pressure on agricultural and processing sectors. The growing need for sustainable, nutritionally balanced feed has spurred greater interest in alternative ingredients for ruminant diets. Camelina (*Camelina sativa* L. Crantz), often referred to as false flax, is an oilseed plant belonging to the *Brassicaceae* family. It can be grown as an annual summer crop or a biennial winter crop (Zubr et al., 2003). Noted for its exceptional adaptability and resilience, camelina thrives in diverse climatic and soil conditions, except for heavy clay and organic soils (Larsson et al*.,* 2013; Avola et al*.,* 2021). In recent years, camelina has attracted growing attention for its adaptability to diverse environmental conditions and its nutritional composition, which is comparable to traditional oilseeds. Its notable resistance to drought, pests, plant diseases, and suitability for rain-fed or dryland farming further highlight its potential as a sustainable agricultural resource (Waraich et al*.,* 2013). The camelina plant is characterized by a stem that may be either smooth or slightly hairy, typically growing to a height of 65–110 cm (Berti et al*.,* 2016). Its reproductive structures, known as camelina seed pods (CSP), are globular siliques partitioned by a central membrane and generally hold between 10 and 25 seeds. During maturation, these pods shift in color from green to yellow-reddish before reaching full dryness (Jankowski et al*.,* 2019). Recent studies have explored the potential of camelina and its derivatives in animal nutrition. Riaz et al. (2022) reported that *C. sativa*, as an oilseed plant, can serve as an effective alternative to traditional protein sources when included in ruminant diets, with minimal adverse effects. Feeding goats with fresh camelina forage has been associated with increased levels of conjugated linoleic acid in milk, alongside a favorable profile of chemical and fatty acid composition, which may benefit human health (Tedone et al*.,* 2022). While prior studies have explored the application of camelina-derived products such as its meal (Lawrence et al*.,* 2016), seeds (Hurtaud & Peyraud., 2007), and forage (Tedone et al*.,* 2022) in ruminant nutrition, there is a notable lack of published research regarding the chemical composition and nutritional value of CSP. However, similar by-products such as rapeseed pod have been reported to possess a high CP content and a low fiber fraction, as documented by Ban et al. (2018). This study seeks to address that gap by evaluating CSP as a potential feed resource, supporting more sustainable livestock production through the use of underutilized agricultural by-products. So, this study aimed to assess the chemical composition, *in vitro* gas production (GP), fermentation parameters, and nutrient digestibility of CSP in comparison to wheat straw (WS) and alfalfa hay (AH). Additionally, the impact of different CSP inclusion levels on diet digestibility and *in vitro* rumen fermentation were determined.

## 2 Materials and methods

### 2.1 Collection of CSP and preparation of experimental diets

The research was conducted in September 2023 at Lorestan University, situated in Khorramabad, Iran. CSP was collected from the Marvdasht district in the Shiraz Province of Iran. During the harvesting of camelina using a combine, the fine seeds and small seed pods often become intermingled due to their size and fineness. After harvesting during the sifting and cleaning process, the CSP were separated from the seeds and then transported to the laboratory for further analysis. The chemical composition and nutritive value of CSP samples were initially analyzed using the *in vitro* GP technique and compared to WS and AH. Then, CSP was incorporated into the dietary forage component at increasing levels of 0 (control), 80, 160 and 240 g/kg DM (Table 1).

**Table 1:** Feed ingredients and chemical composition (g/kg DM or as stated) of the experimental diets containing different levels of camelina seed pods (CSP).

Ingredient, (g/kg DM)	Experimental diets a			
	CSP0	CSP80	CSP160	CSP240
Alfalfa hay	120	80	40	0
Wheat straw	120	80	40	0
Corn, ground	280	280	280	280
Barley, ground	280	280	280	280
Soybean meal	100	100	100	100
Wheat bran	60	60	60	60
Camelina seed pods (CSP)	0.0	80.0	160	240
Calcium carbonate	10.0	10.0	10.0	10.0
Vitamin–mineral premix^b^	14.0	14.0	14.0	14.0
Sodium bicarbonate	12.0	12.0	12.0	12.0
White salt	4.0	4.0	4.0	4.0
Chemical composition				
Dry matter	900	900	900	900
Organic matter	919	921	923	925
Crude protein	140	140	140	140
Rumen undegradable protein (RUP)	57.6	55.6	53.6	51.6
Neutral detergent fiber	300	283	266	249
Lignin	35.5	32.1	29.8	27.8
Ether extract	26.6	25.8	25.0	24.1
Non-fibrous carbohydrate	451	471	491	512
ME (MJ/kg DM)	11.02	11.05	11.09	11.11

ME, metabolizable energy.

a Diets were: Camelina seed pods (CSP) included in the diets at 0 (CSP0), 80 (CSP80), 160 (CSP160) or 240 (CSP240) g/kg dietary dry matter.

b Vitamin–mineral premix Contained per kg: *Ca* 195 g; P 80 g; Mg 21 g; Na 50 g; Fe 3 g; Cu 0.3 g; Zn 0.3 g; Mn 22 g; I 0.12 g; Co 0.1 g; Se 0.02 g; vitamin A 600,000 IU; vitamin D 200,000 IU; vitamin E 200 IU.

### 2.2 Collection of ruminal fluid

Rumen fluid was obtained from two male Lori sheep (average live weight: 40 ± 0.5 kg), each surgically fitted with a permanent rumen cannula. The animals were maintained at the Agricultural Research Station of Lorestan University’s Faculty of Agriculture under conditions aligned with recognized standards for animal welfare. All procedures adhered to the guidelines provided by the Federation of Animal Science Societies for the care and use of agricultural animals in research (FASS, 2010). The sheep received a total mixed ration formulated in accordance with their dietary requirements, as outlined by the NRC (2007). The ration, expressed on a dry matter basis (g/kg DM), consisted of AH (200), WS (300), corn silage (100), wheat bran (120), ground corn (260), slow-release urea (9), dicalcium phosphate (5), sodium bentonite (2.5), and a complex mixture of vitamins, minerals and trace elements (30). The feed was provided in two equal portions each day, at 8:00 a.m. and 6:00 pm., with water accessible at all times. Following a 14-day adaptation period, rumen content was sampled from all quadrants of the rumen prior to the morning feeding. Samples were promptly transferred into a preheated (39 °C) insulated container and transported to the lab. Upon arrival, the samples were homogenized and filtered through triple-layered linen cloth under a CO_2_-rich, anaerobic environment. The time between sampling and incubation was kept within 30 min to ensure microbial viability.

### 2.3 *In vitro* incubation of samples in ruminal fluid

In the initial phase of the experiment, a total of 99 serum bottles were prepared and incubated over three separate experimental runs, each consisting of 33 bottles (comprising three treatments with 10 replicates each, plus 3 blanks). Of these, 36 bottles were allocated for the assessment of *in vitro* gas production (IVGP), while 54 bottles were used to evaluate fermentation characteristics. Additionally, 9 blank bottles were included for baseline correction.

To assess IVGP dynamics, the protocol by Marten and Barnes (1980) was followed. For each incubation, precisely 250 mg of dried, ground sample (particle size: 1 mm) was weighed into 100 mL serum bottles. Each bottle received 5 mL of filtered rumen fluid and 20 mL of buffer solution, then immediately flushed with CO_2_ (oxygen-free), sealed using butyl rubber stoppers and aluminum crimps, gently shaken, and incubated in a water bath maintained at 39 °C. The buffer medium was prepared according to Marten and Barnes (1980), involving the combination of solution A [1 L containing: 10.0 g KH₂PO₄, 0.5 g MgSO₄·7H₂O, 0.5 g NaCl, and 0.1 g CaCl₂·2H₂O] and 20 mL of solution B [prepared as 15.0 g Na₂CO₃ and 1.0 g Na₂S·9H₂O per 100 mL]. The pH was fine-tuned to 6.8 by gradual mixing of solution B into solution A.

Gas production was monitored at multiple time points: 3, 6, 8, 12, 16, 24, 36, 48, 72, and 96 h. The pressure data were converted to gas volumes using a calibration-based linear equation, as suggested by Theodorou et al*.* (1994). After 24 h of incubation, fermentation was terminated by placing the bottles in an ice bath and cooling them gradually to 25 °C. The final gas volume was recorded, and pH was determined using a suitable pH meter (Metrohm 632, Switzerland). For ammonia nitrogen (NH₃-N) analysis, 5 mL of the supernatant was collected from each bottle, acidified with an equal volume of 0.1 N HCl, and stored at –20 °C, following the method described by Broderick and Kang (1980). Residual fermentation solids were dried at 60 °C for 48 h to determine dry matter disappearance (DMD).

### 2.4 *In vitro* fermentation parameter calculations

The kinetics of gas production during *in vitro* fermentation were modeled using the exponential equation proposed by Ørskov and McDonald (1979):

Y=a+b(1−−e−−ct)

where Y represents the gas volume (mL) at incubation time t (h); a is the gas derived from the immediately soluble fraction; b denotes the potential gas production from the insoluble but fermentable fraction; and c is the fractional rate of gas production per hour.

The values for metabolizable energy (ME, MJ/kg DM) and *in vitro* organic matter digestibility (IVOMD) were estimated using equations from Menke et al*.* (1979):

ME = 2.20 + 0.136 × GP + 0.0057 × CP (g/kg DM)
IVOMD = 148.8 + 8.89 × GP + 0.45 × CP (g/kg DM) + 0.65 × ash (g/kg DM)

Here, GP represents net gas production (mL) from 200 mg of dry sample after 24 h of incubation.

Short-chain fatty acid (SCFA) concentrations were estimated following the equation by Getachew et al*.* (2002):

SCFA (mmol/g DM) = 0.0222 × GP − 0.00425

The 24-hour net gas production was expressed as milliliters per gram of dry matter (mL/g DM). Microbial protein (MP) synthesis was calculated using the approach of Blümmel et al*.* (1997):

MP (mg/g DM) = ADS − (GP × 2.2 mg/mL)

where ADS is the apparently digested substrate, and 2.2 mg/mL is a stoichiometric coefficient representing the mass of C, H, and O required for SCFA and gas production per milliliter of gas.

Using data from the initial *in vitro* phase, a second stage was conducted where the forage portion of the diet was gradually substituted with CSP at inclusion levels of 0 (control), 80, 160, and 240 g/kg DM. The same fermentation protocol as stage 1 was used, with the only modification being the number of incubation bottles. A total of 159 bottles were incubated over three runs, each containing 53 bottles (five dietary treatments × 10 replicates + 3 blanks).

### 2.5 Chemical analyses

Samples of CSP, AH, WS, and diets containing varying levels of CSP were analyzed for DM, ether extract (EE), ash and N using standard procedures outlined by AOAC (1995) with procedure number of 930.15, 954.02, 924.05 and 954.01, respectively. The measurement of neutral detergent fiber (NDF), inclusive of residual ash, was performed following Van Soest et al*.* (1991), without incorporating sodium sulfite or amylase in the procedure. Acid detergent fiber (ADF) was determined based on AOAC method 973.18 (AOAC, 1995), with results reported including the ash content. For the determination of acid detergent lignin (ADL), the sulfuric acid-based technique was applied as per Robertson and Van Soest (1981), and values were expressed with residual ash considered. Non-fiber carbohydrates (NFC) were calculated based on Hall's (2000) equation:

NFC (g/kg DM) = 1 – [NDF (g/kg DM) + CP (g/kg DM) + EE (g/kg DM) + ash (g/kg DM)]

Table 1 presents the ingredient and chemical characteristics of the diets formulated with CSP. Calcium was measured by atomic absorption (Perkin elmer, Model PinAAlce 900z, USA), and phosphorus (P) by the vanadate/molybdate (yellow) method (Chapman & Pratt, 1961).

### 2.6 Assay of enzyme activity

Following centrifugation, the precipitated solid fraction was carefully collected to evaluate enzymatic activities associated with particulate matter, based on the procedure outlined by Nogueira Filho et al*.* (2000). The activities of carboxymethyl cellulase (CMCase), microcrystalline cellulase (MCCase), filter paper-degrading enzymes (FPD), α-amylase, and protease were determined using the methodology described by Agarwal (2000).

For CMCase activity, the reaction mixture included 1 mL of 0.1 mol/L phosphate buffer (pH 6.8), 0.5 mL of strained rumen liquor (SRL), and 0.5 mL of a 10 mg/mL carboxymethyl-cellulose solution. The mixture was incubated at 39 °C for 60 min. MCCase activity was evaluated using a similar setup, substituting the substrate with microcrystalline cellulose (10 mg/mL), under identical incubation conditions.

To assess α-amylase activity, a reaction mixture consisting of 1 mL phosphate buffer (0.1 mol/L, pH 6.8), 0.5 mL of SRL, and 0.5 mL of 1 % soluble starch was used. FPD activity was measured using 0.5 g of Whatman No.1 filter paper as the substrate, along with 1 mL of buffer and 1 mL of SRL. All enzymatic reactions were carried out at 39 °C for one hour. Reactions were terminated by the addition of dinitrosalicylic acid (DNS) reagent, and the released glucose was quantified spectrophotometrically according to the method of Miller (1959), using glucose as a calibration standard. Enzyme activities for CMCase, MCCase, FPD, and α-amylase were expressed as µmol of glucose released per mL of reaction mixture per hour.

Protease activity was determined in a separate assay, using a mixture of 1 mL phosphate buffer (0.1 mol/L, pH 6.8), 0.25 mL SRL, and 0.25 mL of casein solution (2.5 mg/mL). The mixture was incubated at 39 °C for 2 h, followed by the addition of 200 mL/L trichloroacetic acid to stop the reaction. The concentration of hydrolyzed protein was determined using the Lowry method (Lowry et al*.,* 1951), and protease activity was expressed as µg of protein degraded per mL per minute.

### 2.7 Statistical analysis

Statistical analyses were carried out using the MIXED procedure in SAS (2001), based on the following model:

Y_ijk_ = µ + T_i_ + R_j_ + e_ijk_,

where Y_ijk_ represents the observed variable, µ is the overall mean, T_i_ denotes the fixed effect of treatment, R_j_ is the random effect associated with each run, and e_ijk_ is the residual error term. Treatment means were separated using Duncan’s multiple range test, with statistical significance considered at *P* < 0.05. To assess the influence of graded levels of CSP inclusion, linear (L) and quadratic (Q) trends were evaluated using orthogonal polynomial contrasts within the SAS environment.

## 3 Results

### 3.1 Chemical composition

The chemical composition of CSP, WS and AH is summarized in Table 2. CSP showed the highest OM content and the lowest ADF levels among the three substrates (*P* < 0.05). Additionally, its crude protein (CP) and NDF content was intermediate between that of WS and AH. CSP had the highest EE and NFC levels compared to the other two forages (*P* < 0.05). The lignin concentration was similar in CSP (67.6, g/kg DM) and AH (79.6, g/kg DM), both of which were lower than in WS (86.2 g/kg DM).

**Table 2:** Chemical composition of camelina seed pods (CSP), wheat straw and alfalfa (g/kg DM or as stated), (*n* = 4).

Parameter	Chemical composition			SEM^b^	*P*-value
	CSP^a^	Alfalfa	Wheat straw		
Dry matter	925	936	942	15.28	0.25
Organic matter	948^a^	900^b^	904^b^	10.7	0.04
Crude protein	93.6^b^	146.0^a^	32.2^c^	0.84	0.01
Neutral detergent fiber	441^b^	408^c^	717^a^	6.56	0.01
Acid detergent fiber	256^c^	336^b^	463^a^	5.65	0.01
Lignin	67.6^b^	79.6^ab^	86.2^a^	2.72	0.02
Ether extract	14.7^a^	11.7^b^	7.35^c^	0.46	0.01
Non-fiber carbohydrates	446^a^	334^b^	147^c^	6.60	0.01
Calcium	2.41^c^	14.7^a^	4.41^b^	0.03	0.01
Phosphorus	0.41^c^	2.72^a^	0.72^b^	0.03	0.01

Means with different letters in a row differ (*P* < 0.05).

a Camelina seed pods.

b SEM, standard error of the mean.

### 3.2 *In vitro* gas production and fermentation parameters

Compared to WS, CSP exhibited significantly greater GP at multiple time points (16, 24, 36, 48, and 72 h of incubation), along with enhanced total GP and a higher estimated GP potential (b). Nevertheless, all GP parameters for CSP remained lower than those observed in AH (*P* < 0.05; Table 3). Moreover, CSP showed significantly higher values of fermentation parameters including IVDMD, IVOMD, ME, MP, SCFA, pH and NH₃-N concentration after 24 h of incubation compared to WS, although these values remained lower than those observed for AH (*P* < 0.05; Table 3).

**Table 3:** *In vitro* gas production and fermentation parameters of camelina seed pods (CSP), wheat straw and alfalfa.

Parameter	Feed ingredients			SEM^b^	*P*-value^c^
	CSP^a^	Alfalfa	Wheat straw		
GP parameters					
GP_16_	25.2^b^	29.3^a^	10.2^c^	0.801	0.01
GP_24_	33.2^b^	39.2^a^	12.9^c^	0.575	0.01
GP_36_	39.6^b^	44.2^a^	20.1^c^	0.599	0.01
GP_48_	46.1^b^	58.3^a^	25.1^c^	0.754	0.01
GP_72_	54.2^b^	65.1^a^	33.1^c^	0.622	0.01
TGP	59.3^b^	69.3^a^	35.5^c^	0.838	0.01
B	61.7^b^	71.1^a^	38.1^c^	1.02	0.01
C	0.054	0.057	0.048	0.004	0.33
Fermentation parameters					
IVDMD ( %)	44.8^b^	53.9^a^	29.9^c^	0.528	0.01
IVOMD ( %)	44.9^b^	55.1^a^	31.8^c^	0.712	0.01
ME (MJ/kg DM)	6.42^b^	7.64^a^	3.80^c^	0.109	0.01
pH	6.37^a^	6.23^b^	6.47^a^	0.035	0.01
Ammonia-N (mg/dL)	12.1^b^	17.9^a^	10.4^c^	0.252	0.01
SCFA (mmol/g DM)	2.22^b^	2.59^a^	0.88^c^	0.071	0.01
MP (mg/g DM)	393^b^	475^a^	276^c^	3.98	0.01

GP_16_, gas production after 16 h incubation; GP_24_, gas production after 24 h incubation; GP_48_, gas production after 48 h incubation; GP_72_, gas production after 72 h incubation; TGP, volume of gas pro-duction (GP) after 96 h of incubation (mL/250 mg sample); b, GP from the fermentable fraction (mL); c, rate constant of GP (mL/h); IVDMD, *in vitro* dry matter (DM) disappearance ( %); IVOMD, *in vitro* organic matter disappearance ( %); ME, estimated metabolizable energy (MJ/kg DM); SCFA, short chain fatty acid (mmol/g DM); MP, microbial protein (mg/g DM);.

a Camelina seed pods.

b SEM, standard error of the mean.

c Means with different letters in a row differ (*P* < 0.05).

Table 4 presents the effects of varying dietary CSP levels on GP parameters. The diet supplemented with CSP240 exhibited the highest GP volume after 16 h of incubation, total GP and GP potential (b), whereas the control diet consistently showed the lowest values for these parameters (L, *P* < 0.05). However, the rate constant of GP (c) remained consistent across all CSP-supplemented diets. Increasing CSP supplementation up to 16 % resulted in a linear improvement in IVOMD, estimated ME, and SCFA production compared to other supplementation levels (L, *P* < 0.05). However, CSP inclusion had no significant effect on pH, IVDMD and NH_3__—_N concentration.

**Table 4:** *In vitro* gas production and fermentation parameters of the experimental diets containing different levels of Camelina seed pods (CSP).

Parameter	Level of CSP in the diet (g/kg DM) a				SEM^b^	*P*-value^c^	
	CSP0	CSP80	CSP160	CSP240		L	Q
GP parameters							
GP_16_	30.3^b^	31.2^b^	31.6^b^	33.7^a^	0.573	0.01	0.32
GP_24_	40.7	42.3	43.3	43.7	1.19	0.09	0.67
GP_36_	50.7	50.9	51.3	52.5	1.13	0.31	0.75
GP_48_	57.7	59.3	59.7	61.5	1.48	0.13	0.87
GP_72_	65.3	65.7	66.5	67.7	1.37	0.24	0.75
TGP	71.9^b^	73.6^ab^	73.9^ab^	76.6^a^	1.39	0.02	0.59
B	73.6^b^	74.3^b^	74.9^b^	78.5^a^	0.767	0.01	0.56
C	0.054	0.056	0.052	0.053	0.0037	0.67	0.87
Fermentation parameters							
IVDMD ( %)	53.1	53.6	54.1	55.3	8.59	0.09	0.66
IVOMD ( %)	53.4^b^	54.1^b^	54.3^ab^	55.9^a^	5.11	0.01	0.35
ME (MJ/kg DM)	7.71^b^	7.81^b^	7.88^b^	8.15^a^	0.078	0.01	0.34
pH	6.45	6.39	6.37	6.31	0.051	0.09	0.97
Ammonia-N (mg/dL)	17.5^a^	18.3^ab^	18.5^ab^	18.9^a^	0.353	0.02	0.61
SCFA (mmol/g DM)	2.68^b^	2.75^b^	2.79^b^	2.97^a^	0.051	0.01	0.32
MP (mg/g DM)	464	467	471	479	8.42	0.23	0.77

GP_16_, gas production after 16 h incubation; GP_24_, gas production after 24 h incubation; GP_48_, gas production after 48 h incubation; GP_72_, gas production after 72 h incubation; TGP, volume of gas production (GP) after 96 h of incubation (mL/250 mg sample); b, GP from the fermentable fraction (mL); c, rate constant of GP (mL/h); IVDMD, *in vitro* dry matter (DM) disappearance ( %); IVOMD, *in vitro* organic matter disappearance ( %); ME, estimated metabolisable energy (MJ/kg DM); SCFA, short chain fatty acid (mmol/g DM); MP, microbial protein (mg/g DM); Means with different letters in a row differ (*P* < 0.05).

a Diets were: camelina seed pods (CSP) included in the diet at 0 (CSP0), 80 (CSP80), 160 (CSP160) or 240 (CSP240) g/kg dietary dry matter.

b SEM, standard error of the mean.

c L, Linear; Q, Quadratic.

### 3.3 Rumen enzymatic activity

Fibrolytic enzyme activities, including CMCase, increased linearly (L, *P* < 0.05) with elevated levels of CSP in the diet, while MCCase demonstrated a tendency for linear improvement (L, *P* = 0.07). Additionally, both α-amylase and protease activities exhibited a significant linear increase (L, *P* < 0.05) in response to higher dietary inclusion of CSP (Table 5).

**Table 5:** Hydrolytic enzyme activity in the rumen (U/h/mL or as stated) in response to experimental diets containing varying levels of camelina seed pods (CSP).

Parameter	Level of CSP in the diet (g/kg DM) a				SEM^b^	*P*-value^c^	
	CSP0	CSP80	CSP160	CSP240		L	Q
Carboxymethyl cellulase	33.2^b^	34.3^ab^	35.1^ab^	37.3^a^	1.02	0.02	0.67
Microcrystalline cellulas	15.3	16.4	16.7	17.7	0.738	0.07	0.91
Filter paper degrading	24.2	23.3	24.3	25.3	1.02	0.35	0.42
ɑ-amylase	75.6^b^	79.3^ab^	80.3^ab^	82.3^a^	1.48	0.01	0.59
Protease (μg hydrolyzed protein/mL/min)	170^c^	174^c^	181^b^	189^a^	1.83	0.01	0.38

Means with different letters in a row differ (*P* < 0.05).

a Diets were: camelina seed pods (CSP) included in the diet at 0 (CSP0), 80 (CSP80), 160 (CSP160) or 240 (CSP240) g/kg dietary dry matter.

b SEM, standard error of the mean.

c L, Linear; Q, Quadratic.

## 4 Discussion

Drawing upon existing knowledge, this study addresses a novel area within the field, for which no direct references or prior investigations are currently available. As a result, identifying comparable studies to substantiate our findings posed a considerable challenge, given that this research represents the first known examination of this specific subject. Nevertheless, the study is guided by recent advancements in the utilization of various seed pods—such as those from soybean, rapeseed, pea, and faba bean in livestock nutrition, which highlight the broader potential of agricultural by-products as sustainable feed resources. The chemical composition and other nutritional parameters, including IVGP and fermentation characteristics of CSP highlight its potential as an alternative feed source for ruminants. Its CP content falls within an acceptable range for livestock nutrition, exceeding that of WS while being lower than that of AH. In most aspects, except for CP content, its nutritional value surpasses that of both compared forages. For instance, CSP contains higher levels of OM, NFC, and EE than WS and AH, while its ADF and lignin content are lower than those of both forages. According to Sruamsiri and Silman (2008), soybean pod husks contain moderate nutritional value, with CP, EE, NDF, and ADF reported at 5.05, 1.65, 60.15, and 42.08 g/100 g DM, respectively. Content of ADF is widely recognized as a critical determinant of forage digestibility, with higher ADF levels generally associated with reduced digestibility and, consequently, lower energy availability for animals (Schick & Beckman, 2023). Among the components contributing to high ADF, lignin plays a particularly inhibitory role in ruminal digestion. It forms chemical bonds with structural carbohydrates and serves as a physical shield, limiting microbial access to plant cell walls (Raffrenato et al*.,* 2017). Additionally, research by Mateos-Aparicio et al*.* (2010) indicated that pea and broad bean pods offer higher protein content, with crude protein concentrations of 10.8 and 13.6 g/100 g DM, respectively. Tedone et al*.* (2022) analyzed the chemical composition of camelina forage at different growth phases, reporting average values of 23.2 % DM, 15.91 % CP, 8.49 % ash, and 3.27 % EE. These compositional metrics exhibit marked differences when compared to the nutritional characteristics of CSP identified in the current investigation. Variations in GP are indicative of differences in the chemical composition of feed ingredients and are commonly used to estimate their nutritive value. Furthermore, GP also reflects the formation of SCFA and MP, as noted by Kazemi et al. (2021). Throughout the fermentation process, microbial breakdown of carbohydrates and proteins results in the formation of metabolites like acetate, butyrate, and ammonia, which are associated with GP. The enhanced GP and fermentation efficiency observed for CSP compared to WS are likely due to differences in their chemical compositions. Due to its lower levels of ADF, NDF, and lignin, corn CSP have a more accessible and easily fermentable cell wall structure. This facilitates the degradation process and increases the availability of fermentable substrates for cellulolytic microorganisms, thereby supporting their growth and activity (Weimer, 2022). The proliferation of these microbes plays a critical role in determining the extent and rate of substrate degradation, which is directly linked to the volume of GP during fermentation (Blümmel et al*.,* 1997). The relatively accessible nutrient profile of CSP may foster enhanced microbial proliferation and metabolic activity within the rumen. Nonetheless, despite some improvements in fermentation responses, GP values for CSP remained significantly lower than those of AH, a benchmark high-quality forage in ruminant diets. This discrepancy underscores CSP’s potential as an alternative feed resource, while also highlighting its limitations in fermentation efficiency relative to conventional, highly digestible forages such as AH. Supporting this, Sruamsiri and Silman (2008) reported that soybean pod husks yielded higher levels of total digestible nutrients and digestible energy compared to guinea grass. Likewise, rapeseed pod meal was shown to improve rumen fermentation and increase IVDMD, reinforcing its suitability as a concentrate substitute (Wanapat et al*.,* 2020). CSP's lower gas production potential (parameter b) relative to AH could be attributed to its higher fiber content, which may reduce microbial access and fermentation efficiency (Mailer et al*.,* 2008).

In addition to GP, CSP demonstrated superior performance compared to WS in multiple fermentation parameters, including IVDMD, IVOMD, ME, MP synthesis, SCFA production, pH, and NH_3__—_N concentration after 24 h of incubation. These outcomes align with previous research showing that oilseed by-products, due to their higher nutrient density, often support better digestibility and microbial activity compared to more fibrous crop residues like WS (Sergheeva & Netreba, 2024). The higher levels of IVDMD and IVOMD for CSP are particularly noteworthy, as these values are important indicators of the feed's energy potential and nutritional value for ruminants.

Njidda and Oloche (2022) reported a positive correlation between CP content and IVDMD, as well as a negative correlation between IVDMD and cell wall components during their evaluation of the chemical composition and digestibility of leaves from semi-arid browse species in North-East Nigeria. These findings are consistent with our results, where a similar pattern was observed for IVDMD in relation to the cell wall and CP content of CSP compared to WS and AH.

The superior IVDMD, IVOMD and SCFA production observed with CSP compared to WS can be attributed to its enhanced fermentability, as evidenced by its higher GP volume. Earlier research has indicated that a rise in asymptotic GP is associated with enhanced DM digestibility, as gas is produced during the microbial fermentation of carbohydrates into volatile fatty acids such as butyrate and acetate (Kazemi et al., 2021). Moreover, the greater GP, enhanced fermentation characteristics, and better nutrient digestibility observed in AH relative to other experimental feeds like CSP and WS are probably attributable to its higher CP content (Azizi et al*.,* 2020). Similarly, Oni et al*.* (2010) observed a significant positive correlation between the CP content of cassava leaves and the volume of GP after 48 h of incubation, suggesting that higher protein levels may enhance microbial fermentation and gas production efficiency. Furthermore, the elevated NH_3__—_N concentration observed with the incubation of CSP or AH, compared to WS, is likely a result of their higher CP levels, which are more readily degraded by rumen proteolytic microbes. It has been noted that both the protein structure and the concentration of protein in the diet are crucial factors influencing its susceptibility to microbial proteases in the rumen and, consequently, its degradability (Bach et al*.,* 2005). Nevertheless, despite these promising fermentation results, CSP exhibited lower fermentation parameters compared to AH. This suggests that while CSP may be a valuable supplement to traditional feed ingredients, it may need to be combined with higher-quality forages or further processed to enhance its nutritional profile and fermentation efficiency for optimal use in ruminant diets.

The data reveals that the diet supplemented with CSP240 exhibited the highest GP volume after 16 h of incubation, total GP, and GP potential (b), while the lowest values were observed in control diet. This indicates that increasing the CSP content in the diet positively influences the overall fermentability, as reflected by enhanced GP. These findings align with the observed increase in OM and NFC, along with the reduction in NDF and lignin content, as the level of CSP in the diet increases. This pattern is consistently associated with enhanced digestibility of ruminant diet. Getachew et al*.* (2004) and De Boever et al*.* (2005) demonstrated a positive association between NFC and both the potential GP and gas volumes measured at 6, 24 and 48 h of incubation. In contrast, the negative correlations observed between NDF, ADF and ADL contents and IVGP across different incubation times are consistent with the results reported by Gasmi-Boubaker et al. (2005) and Sallam et al. (2007), highlighting the inhibitory effect of structural carbohydrates on fermentative activity.

Despite the improvements in GP, the c remained constant across all CSP-supplemented diets. This observation suggests that while higher levels of CSP may improve the overall GP and GP potential, it could not alter the rate at which fermentation occurs. It is possible that microbial adaptation to higher levels of CSP may result in more efficient fermentation over time without a significant change in the speed of the process.

Furthermore, increasing CSP supplementation up to 16 % resulted in a linear improvement in IVOMD, ME and SCFA production compared to lower supplementation levels. These results support the hypothesis that enhancing the nutritional profile of ruminant diets can improve digestibility and energy yield. The linear increase in SCFA production with higher CSP supplementation is particularly significant, as SCFAs play a crucial role in maintaining rumen health and providing energy to ruminants. Melesse (2012) indicated that SCFA content is a key indicator of the energy value of diets. Similarly, Getachew et al*.* (2002) observed a strong correlation between SCFA production and IVGP, utilizing this relationship to estimate SCFA production based on *in vitro* gas values. Furthermore, the observed increase in ME suggests that higher CSP levels enhance energy utilization efficiency, which could have practical implications for livestock production, particularly in improving animal production efficiency (Makkar & Beever, 2013).

The observed linear increase in the activity of CMCase with higher CSP levels suggests that CSP supplementation positively influences fibrolytic enzyme activity. This enhancement is likely due to the increased availability of fermentable carbohydrates, which serve as substrates for cellulolytic microorganisms in the rumen. Additionally, the tendency for a linear improvement in MCCase activity further supports the notion that CSP enhances fiber digestibility by promoting the activity of multiple fibrolytic enzymes. The increase in α-amylase and protease activities with higher CSP supplementation also indicates that CSP may have a broader impact on enzymatic digestion beyond fiber degradation. α-amylase plays a crucial role in starch hydrolysis (Radzlin et al., 2024), while proteases break down proteins into amino acids and peptides, which are essential for growth and maintenance in ruminants. The positive effects of CSP on both α-amylase and protease activities suggest that CSP may improve the overall digestive capacity of ruminants, particularly in the breakdown of non-fibrous carbohydrates and proteins. Azizi-Shotorkhoft et al*.* (2016) showed that bacteria with amylolytic and cellulolytic activity quickly colonize carbohydrates that are soluble and readily degradable. This early colonization increases the availability of targeted substrates, which in turn facilitates the subsequent proliferation of other fibrolytic microbial populations. The results from this study also suggest that CSP supplementation has the potential to improve overall feed efficiency in ruminants by enhancing enzyme activity associated with the digestion of both fibrous and non-fibrous feed components. Increased enzyme activity, particularly those involved in fiber degradation and starch and protein hydrolysis, could lead to better nutrient absorption and, consequently, improved animal performance.

## 5 Conclusion

The results of present study reveal that camelina seed pods possess favorable chemical and nutritional characteristics, positioning it as a viable alternative forage source in ruminant diets. Specifically, camelina seed pods demonstrated superior organic matter content, higher gas production, and improved nutrient digestibility and fermentation efficiency compared to wheat straw. Additionally, several fermentation parameters of CSP were comparable to those observed with alfalfa hay. The observed linear increases in fibrolytic enzyme activity and key fermentation traits with increasing levels of CSP inclusion further indicate its capacity to enhance rumen microbial function and overall nutrient utilization. These findings suggest that camelina seed pods not only meets basic nutritional requirements but also offers functional benefits for digestive efficiency, thereby justifying its use as a forage substitute. Finally, in order to validate the findings of the current study, it is imperative that animal experiments be conducted.

## Ethical statement

All experimental procedures involving animals were conducted in accordance with the guidelines established by the Federation of Animal Science Societies (FASS, 2010) for the care and use of agricultural animals in research. The study protocol was reviewed and approved by the Institutional Animal Care and Use Committee of Lorestan University’s Faculty of Agriculture, ensuring compliance with ethical standards for animal welfare.

Ayob Azizi

Associate Professor, Department of Animal Science, Faculty of Agriculture, Lorestan University, Khorramabad, Iran.

## CRediT authorship contribution statement

**Alireza Jolazadeh:** Writing – review & editing, Writing – original draft, Supervision, Methodology, Funding acquisition, Conceptualization. **Ayoub Azizi:** Project administration, Methodology, Funding acquisition, Formal analysis. **Afrooz Sharifi:** Writing – review & editing, Formal analysis. **Fatemeh Memarzadeh:** Methodology. **Nazanin Fallali:** Methodology. **Elham Imani-Rad:** Methodology.

## Declaration of competing interest

The authors declare that they have no known competing financial interests or personal relationships that could have appeared to influence the work reported in this paper.
